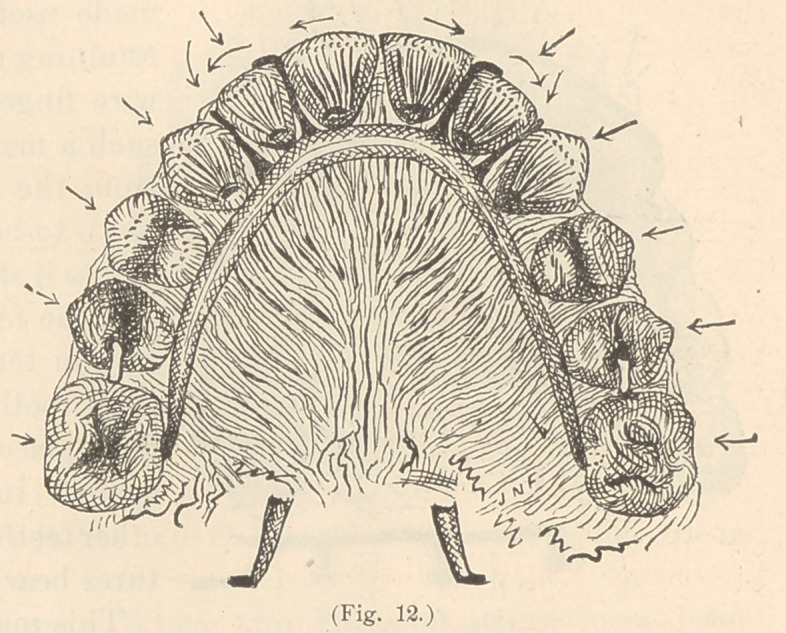# The Uses of Ferrules in Regulating Teeth

**Published:** 1886-07

**Authors:** J. N. Farrar

**Affiliations:** New York City


### Independent Practitioner.

VOL. VII. JULY, 1886. NO. 7.

### Original Communications.

NOTE.—No paper published or to be published in another journal will be accepted for this department. All papers must be in the hands of the Editor before the first day of the month preceding that in which they are expected to appear. Extra copies will be furnished to each contributor of an accepted original article, and reprints, in pamphlet form, may be had at the cost of the paper, press-work and binding, if ordered when the manuscript is forwarded. The Editor and Publishers are not responsible for the opinions expressed by contributors. The journal is issued promptly, on the first day of each month.

### THE USES OF FERRULES IN REGULATING TEETH.

(ROTATING, DRAWING AND RETAINING DEVICES.)

BY J. N. FARRAR, M. D., D. D. S., NEW YORK CITY.

AN EXTRACT FROM AN ILLUSTRATED LECTURE, DELIVERED BEFORE THE MASS. STATE DENTAL SOCIETY, AT BOSTON, DECEMBER 11, 1885.

*Rotators:*—Rotating apparatus consists of two requisites; the instrument of force and the means of attachment. These may be combined in one or in more pieces. While the former has been considered comparatively easy to devise, the means of firmly connecting it to the tooth has been regarded as quite the contrary. To overcome this difficulty I have devised several fixtures more or less complicated, some of which are simple and easy to operate, but most of them are practical only in the hands of experts. Since the advent of quick-setting cements, however, I have been able to increase the number of the simpler class through the aid of ferrules, which make the firm attachment of any instrument of force possible, under almost any circumstances.

Ferrules used to permanently bind broken and decayed teeth, subsequently filled with amalgam or other cement, have been in vogue for a long time; but the use of the ferrule, as an instrument for the purpose of turning teeth, by the aid of a lever connected with it, is of a much later origin. When and by whom this was first attempted, I know not. Several years ago Dr. Atkinson mentioned having made something of the kind in the form of a long-handled dipper; but until the introduction into dentistry of phosphate of zinc made it possible to fix ferrules upon the teeth quickly, the idea was not considered very practical.

(Fig. 1.)

The principle of construction and application of all such devices is as follows: A strip of pure gold plate (No. 30), cut long enough to band the tooth and overlap a trifle, is rolled and tied with steel hair-wire to hold the lap in place; after which it is laid in a wire nest, having a handle made of the same material. Having then applied borax and solder, it is held in the flame of an *ordinary office* spirit *lamp.*

For the lodgment of the point of a jack-screw, a hole drilled through the ferrule is often sufficient; but when a deeper pit is necessary, a socket may be attached with solder; wire, however, is more useful.

(Fig. 2.)

When such auxiliaries as rings or staples are desired, a hole is drilled of the size of the wire. In each of these holes is then placed one end of the wire, to fix it in place while being soldered.

(Fig. 3.)

Instead of gold wire, I generally prefer platinum for levers, staples, etc., because being more pliable and less elastic its form is more easily changed, if required during the operation. Of course the degree of stiffness in these wires is governed by the quantity of metal.

This quantity may be embodied in a single wire, or in smaller wires doubled and twisted. When a loop at the end is needed for attachment to other things, the latter is often preferable.

(Fig. 4.)

When all is ready, the ferrule is placed upon the tooth and the wire bent to the proper form, after which the ferrule is cemented to the tooth with phosphate of zinc, while of a *sticky* consistency. After the cement has hardened the ferrule portion of the apparatus may be relied upon for "wrench forces," or for attachment of any desired instrument of force.

(Fig. 5.)

One of the simplest methods of turning a tooth set in a ferrule having a platinum wire lever, is by bending the wire from day to day so that it will firmly bear upon an adjacent tooth in such a manner as to lift upon the ferruled tooth, (Fig. 1.) This, however, requires great care. In order to avoid causing pain, the ferrule end of the lever should be firmly held with pliers while it is being bent at the other end by another pair.

(Fig. 6.)

Fig. 2 illustrates one of my favorite methods of turning two lateral incisors at the same time, with one jack-screw. To prevent the teeth from being driven outside of the esthetic line of the dental arch by the force of the screw, they are tied with a string or small wire passed through staples soldered to the labial surfaces of the ferrules, as shown. The lower portion of the figure illustrates the apparatus in detail.

(Fig. 7.)

Fig. 3 illustrates one of my new forms of jack-screws improvised by necessity for this purpose. As will be seen, it is simply one of my triplex acting screws, with a nut. Each end serves as a spindle point.

In these double cases, where one tooth requires a greater degree of rotation than the other, the differences may be balanced by corresponding differences in the length of the levers. A lever may be an inch in length, or it may be only a staple.

Fig. 4 illustrates, on the right side, a simple device for moving a lateral incisor from the posterior position into line, by means of a wire soldered to the labial surface of a ferrule. The operation consists in bending the wire from day to day until it is straight, as shown on the left. There is sometimes a drawback in this method, through an adverse movement of the tooth or teeth upon which the lever bears, but such teeth will correct themselves upon regaining their liberty. When the tooth is regulated the same device may serve as a retainer. This leads me to the consideration of retainers in general.

(Fig. 8.)

*Retaining Apparatus.*—When two lateral incisors have been forced into position (Fig. 2), they may be temporarily retained very easily by a wire through staples soldered on the labial surfaces of the ferrules, so that the wire will rest upon the anterior surfaces of adjacent teeth, as shown in Fig. 5. I have, by the same means, drawn instanding laterals into line by the elasticity of the same device, when they did not need turning, but this is generally a painful process. (Fig. 6.)

Fig. 7 illustrates a modification of this, which consists in retaining the teeth independently by a shorter piece of wire, extending each way from the staple, so as to rest upon the opposite adjacent teeth. Both of these devices, however, are so unsightly that I never use them, except as a temporary resort, while other teeth are being regulated.

(Fig. 9.)

A ferrule may sometimes be made useful as a portion of a retaining apparatus, by having wire fingers soldered to it in such a manner as to reach out from the ferrule against the tooth to be held in place. Of course it should be remembered that the force upon the finger, by the tendency in the regulated tooth to return to its former place, must be guarded against in order to prevent other teeth, upon which the fixtures bear, from moving.

(Fig. 10.)

This may easily be obviated by other fingers to bear elsewhere. For instance, to retain in position a lateral incisor that has been moved into line from an instanding position, by a wire finger extending from a ferrule placed on the first bicuspid and projecting forward, escaping the lingual surface of the cuspid to bear upon the incisor, there should be a branch wire to bear on the labial surface of the cuspid, as shown in Fig. 8.

(Fig. 11.)

If the ferrule is properly fitted and set, it may remain many months without harm, like a gold thimble crown. My favorite method of retaining teeth, however, when practicable, is by the use of wires without ferrules, bent into the proper shape, one end to bear upon the regulated tooth and the other anchored in a cavity elsewhere with phosphate of zinc or amalgam.

To give greater firmness of anchorage, a branch wire should extend into another tooth. One form of this device is illustrated in the same figure.

(Fig. 12.)

Both of these principles are applicable in retaining *protruding* front teeth that have been *regulated.* As an illustration: A wire having T's to rest between the incisors to prevent it from impinging upon the gum, may extend around in front, inside or out, and be anchored in ferrules or in cavities in the sides of the molars, or in the grinding surfaces of the bicuspids and molars. When ferrules or shell-crowns are used, the wires may be fastened to them with solder, or they may pass through smaller ferrules soldered to the larger, and the wire fixed in place by small thumb-screws or nuts, through or behind the smaller ferrules. These wires may be bent variously as shown in Fig. 9, but the strongest plan is as illustrated by Fig. 10.

To retain teeth after the *enlargement* of the dental arch, instead of the old-fashioned roof-plate, a skeleton device of half round gold wire is preferred. Several modifications of this are shown in Fig. 11.

Fig. 12 illustrates one of my favorite varieties of this class of fixtures for retaining such teeth. On the end of each leg is a pin point which rests in a pit in a molar tooth, and for holding in position rotated lateral incisors by means of delicate spurs of clasp metal. These pits I often make previously, for retaining regulating apparatus in position.

The arrows on these several figures show the direction of the tendency of the regulated teeth to return. The number and form of smaller retainers, which I have devised, are numerous; several are shown in Fig. 10, all of which are so plainly illustrated that they need no further explanation.

## Figures and Tables

**Fig. 1. f1:**
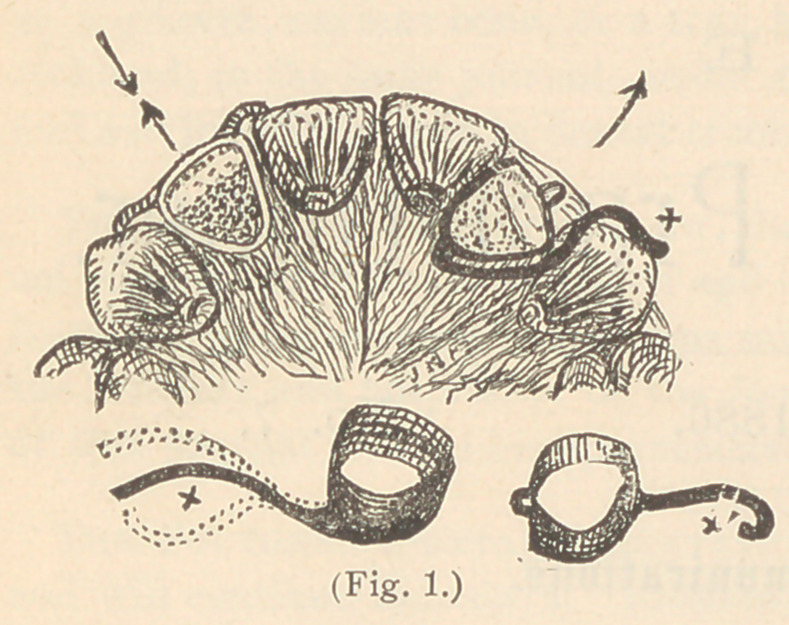


**Fig. 2. f2:**
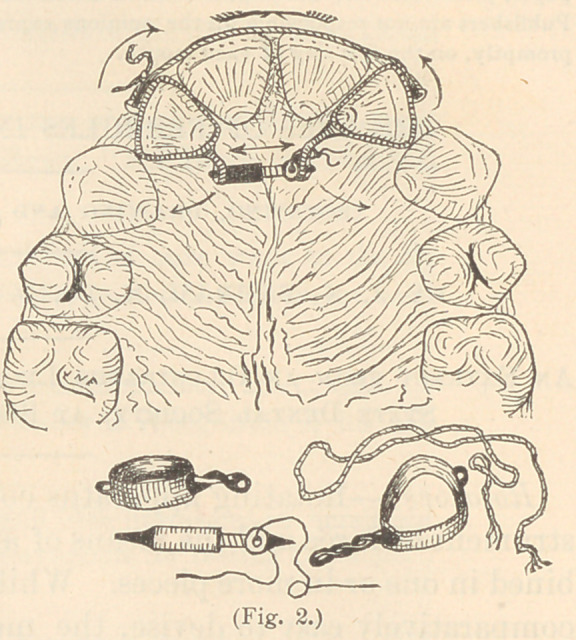


**Fig. 3. f3:**
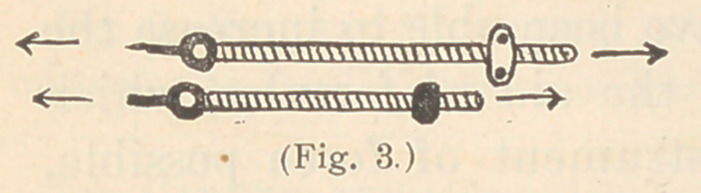


**Fig. 4. f4:**
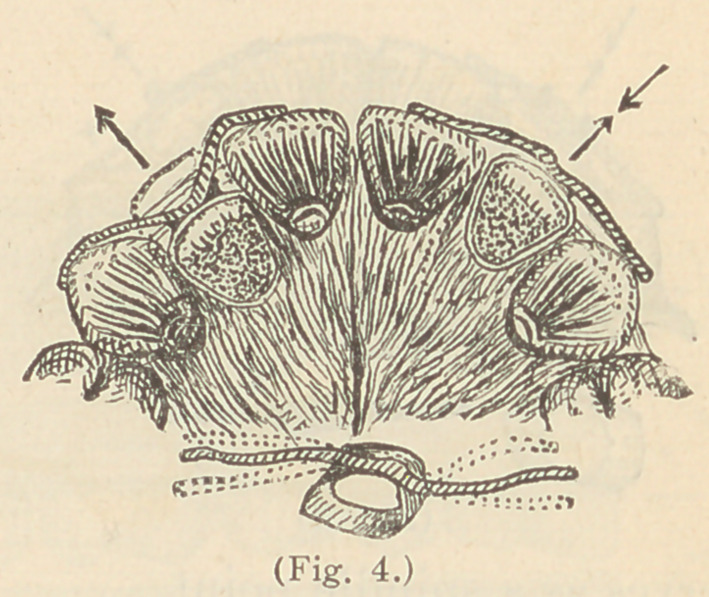


**Fig. 5. f5:**
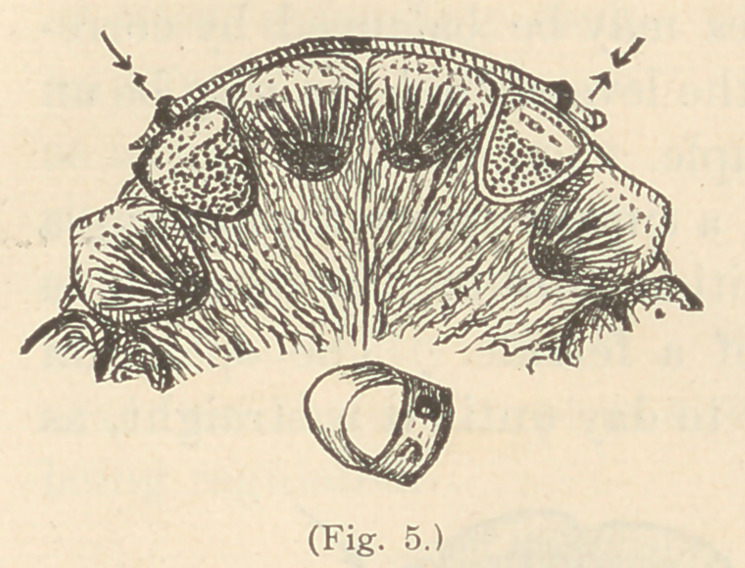


**Fig. 6. f6:**
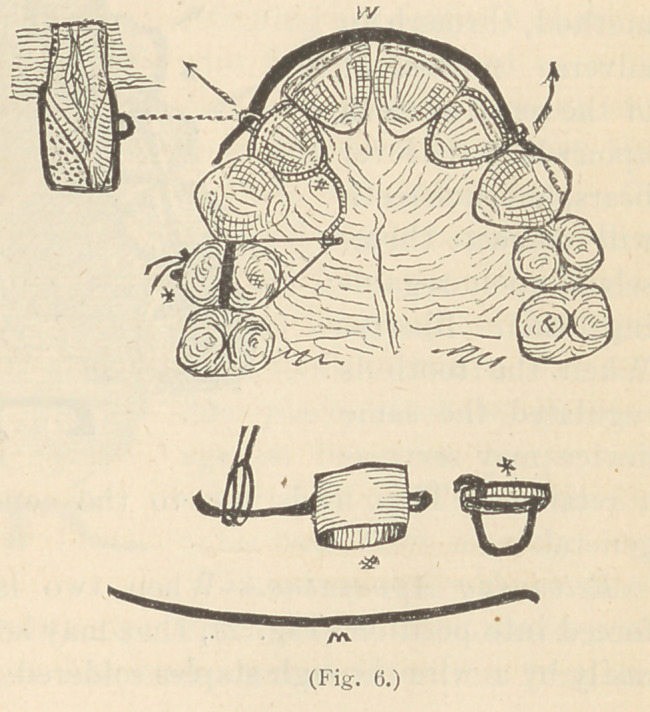


**Fig. 7. f7:**
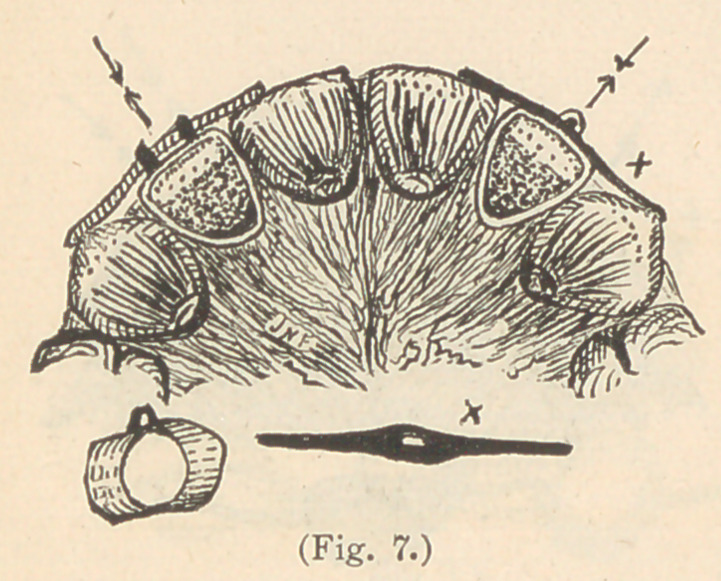


**Fig. 8. f8:**
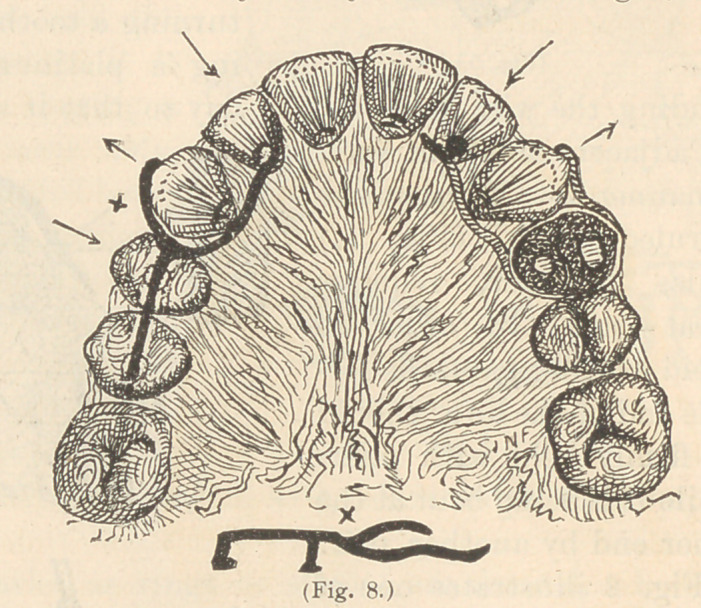


**Fig. 9. f9:**
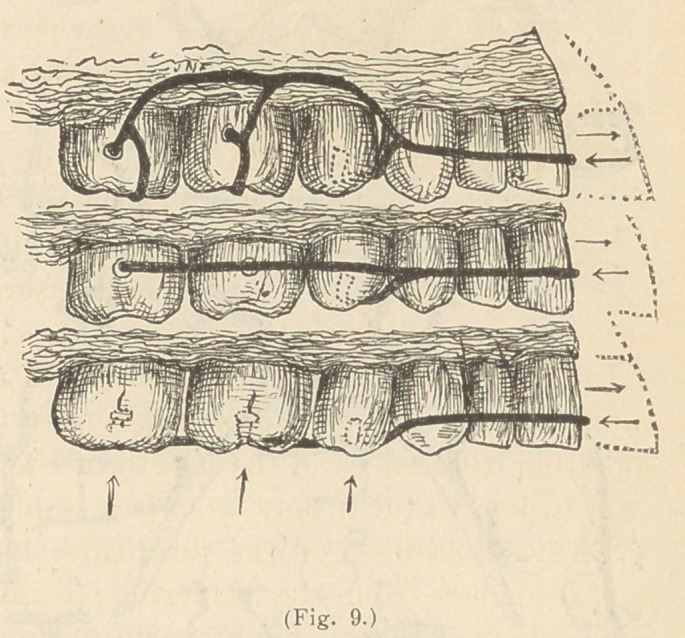


**Fig. 10. f10:**
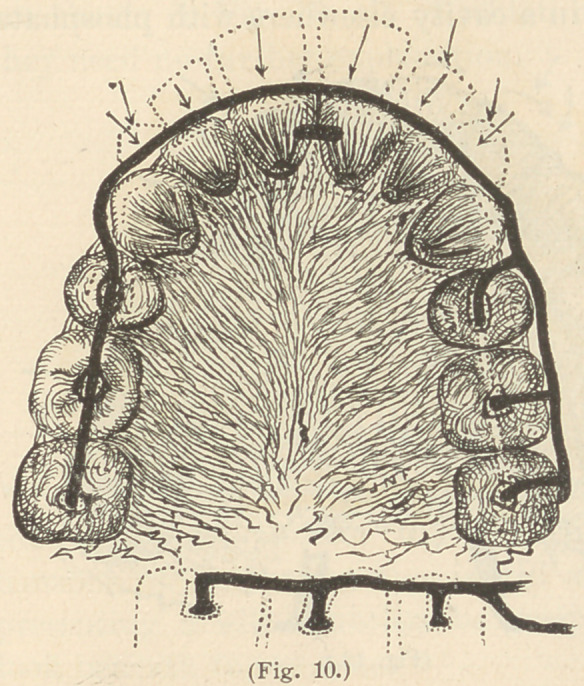


**Fig. 11. f11:**
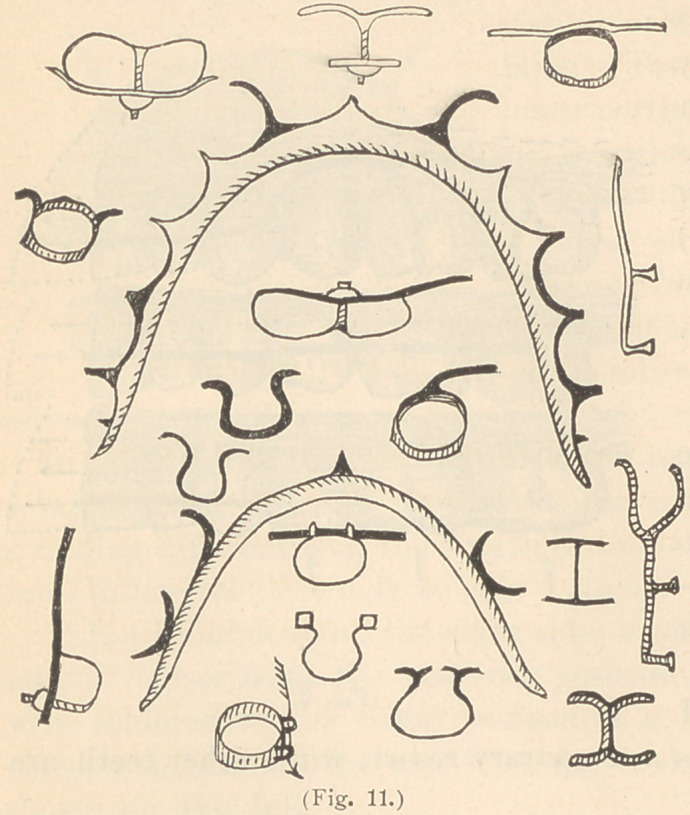


**Fig. 12. f12:**